# Age-Related Deterioration of Perineuronal Nets in the Primary Auditory Cortex of Mice

**DOI:** 10.3389/fnagi.2016.00270

**Published:** 2016-11-08

**Authors:** Dustin H. Brewton, Jamiela Kokash, Oliva Jimenez, Eloy R. Pena, Khaleel A. Razak

**Affiliations:** ^1^Graduate Neuroscience Program, University of CaliforniaRiverside, CA, USA; ^2^Department of Psychology, University of CaliforniaRiverside, CA, USA

**Keywords:** auditory cortex, aging, hearing loss, inhibition, extracellular matrix, perineuronal nets

## Abstract

Age-related changes in inhibitory neurotransmission in sensory cortex may underlie deficits in sensory function. Perineuronal nets (PNNs) are extracellular matrix components that ensheath some inhibitory neurons, particularly parvalbumin positive (PV+) interneurons. PNNs may protect PV+ cells from oxidative stress and help establish their rapid spiking properties. Although PNN expression has been well characterized during development, possible changes in aging sensory cortex have not been investigated. Here we tested the hypothesis that PNN+, PV+ and PV/PNN co-localized cell densities decline with age in the primary auditory cortex (A1). This hypothesis was tested using immunohistochemistry in two strains of mice (C57BL/6 and CBA/CaJ) with different susceptibility to age-related hearing loss and at three different age ranges (1–3, 6–8 and 14–24 months old). We report that PNN+ and PV/PNN co-localized cell densities decline significantly with age in A1 in both mouse strains. In the PNN+ cells that remain in the old group, the intensity of PNN staining is reduced in the C57 strain, but not the CBA strain. PV+ cell density also declines only in the C57, but not the CBA, mouse suggesting a potential exacerbation of age-effects by hearing loss in the PV/PNN system. Taken together, these data suggest that PNN deterioration may be a key component of altered inhibition in the aging sensory cortex, that may lead to altered synaptic function, susceptibility to oxidative stress and processing deficits.

## Introduction

Age-related decline in sensory function affects the quality of life in the elderly (Carabellese et al., 1993; Dalton et al., 2003; Guthrie et al., 2016). In the auditory realm, this is exemplified by the relationship between age-related hearing loss (presbycusis), speech recognition problems, social isolation, depression and cognitive decline (Weinstein and Ventry, 1982; Gordon-Salant et al., 2006; Panza et al., 2015; Wayne and Johnsrude, 2015; Deal et al., 2016; Peelle and Wingfield, 2016). Presbycusis affects ~35% of humans older than 65 and ~45% of humans older than 75 years (Gates and Mills, 2005). Decline in sensory function in presbycusis likely reflects a combination of peripheral sensory organ deterioration and central aging (Frisina and Frisina, 1997; Syka, 2010). The relative contributions of these factors to auditory processing deficits with presbycusis remain unclear.

A consistent finding across the sensory cortex is a decline in inhibitory neurotransmission with age (Leventhal et al., 2003; Caspary et al., 2008; Syka, 2010; Hickmott and Dinse, 2013). Processing of rapidly changing spectrotemporal cues, such as those present in speech, depends on interactions between excitatory and inhibitory components of the auditory neuron receptive field (Zhang et al., 2003; Razak and Fuzessery, 2006, 2008, 2009; Sadagopan and Wang, 2010; Trujillo et al., 2013). Reduced inhibition may, therefore, affect temporal and spectrotemporal processing, and cause speech processing deficits with age (Walton et al., 1998; Mendelson and Ricketts, 2001; Parthasarathy and Bartlett, 2011; Suta et al., 2011; Trujillo and Razak, 2013). The cellular correlates of reduced inhibition in the auditory system remain incompletely characterized.

Of particular relevance to selectivity for fast spectrotemporal changes are rapid spiking cortical interneurons that express the calcium buffering protein, parvalbumin (PV; Atencio and Schreiner, 2008; Moore and Wehr, 2013). These cells may shape inhibition in auditory cortical neurons that is necessary for some forms of spectrotemporal selectivity (Wu et al., 2008). The number of PV+ cells in the primary auditory cortex (A1) declines with presbycusis (de Villers-Sidani et al., 2010; Martin del Campo et al., 2012; Ouellet and de Villers-Sidani, 2014; Ouda et al., 2015) suggesting a cellular correlate of presbycusis-related decline in spectrotemporal processing.

The mechanisms underlying age-related reduction in the number of PV+ cells remain unclear, but recent studies suggest that perineuronal nets (PNN) may play a major role. Many PV+ cells in A1 are ensheathed by PNN (Happel et al., 2014; Fader et al., 2016). PNNs are specialized extracellular matrix components and consist of chondroitin sulfate proteoglycans (CSPG) that are found throughout the extracellular matrix, but are highly dense around PV+ interneurons. PNNs may protect PV+ cells from oxidative stress that accumulate with age (Cabungcal et al., 2013; Suttkus et al., 2016). However, relatively little is known about changes to PNN with age in any sensory cortex. Therefore, the main aim of this study was to test the hypothesis that PNN deteriorates with age in A1. Because of the association between PV and PNN, we also examined if PV+, PNN+ and PV/PNN co-localized cell densities changed with age. We examined PV and PNN expression in two mouse strains. The C57BL/6 (C57) is a model for presbycusis with significant hearing deficits due to hair cell loss that begins at ~3 months of age (Willott et al., 1993; Spongr et al., 1997; Zheng et al., 1999). The CBA/CaJ (CBA) mouse does not show significant hair cell loss with age even up to 18 months of age. Within the constraints imposed by differences in genetic backgrounds, comparison of these two strains will indicate if there is an overall age-related decline in PV, PNN and PV/PNN expression in A1 and if peripheral hearing loss exacerbates such a decline. The data show an age-related decline in PNN+ cell and PV/PNN co-localized cell density in both strains. However, an age-related decline in PV+ cell density occurred only in the C57 mice. Moreover, in the cells that remain PNN+ in the old mice, there is a reduction in PNN intensity only in the C57 mice. Overall, these results suggest that changes in PNN and PV expression with age likely underlie altered inhibition in sensory cortex.

## Materials and Methods

### Experimental Groups

All the mice used in this study were obtained from breeding triads purchased from Jackson Laboratories (Bar Harbor, ME, USA). Littermates were housed in cages of 2–4 individuals on a 12:12 h light-dark cycle and fed *ad libitum*. Mice were housed and raised in the same vivarium under similar conditions. Data were collected from three age groups: young (“Y”, 1–3 months old), middle aged (“M”, 6–8 months old), and old (“O”, 14–24 months old). The institutional Animal Care and Use Committee at the University of California, Riverside approved all procedures.

### Immunohistochemistry

All steps were performed at room temperature unless stated otherwise. Mice were injected with a lethal dosage of sodium pentobarbital and then transcardially perfused using a peristaltic pump (Harvard Apparatus, MA, USA) with 0.1 M phosphate buffer saline (PBS) followed by 4% paraformaldehyde (PFA, pH 7.4). Brains were immediately removed and post-fixed in 4% PFA for 2 h at 4°C. Following post-fixation, brains were placed in a 30% sucrose solution until they sunk (~36–48 h at 4°C). Brains were sectioned into 40 μm slices on a cryostat (CM1860, Leica, IL, UK) and stored in a 0.1 M PBS solution containing 0.1% sodium azide. Immunohistochemistry was performed on free-floating sections with agitation at room temperature, unless otherwise specified. Sections were pre-treated with 4% PFA for 2 h, then rinsed for 5 min with 0.1 M PBS solution two times. Sections were then treated with 50 mM ammonium chloride for 15 min, followed by three rinses in 0.1 M PBS for 5 min each. Sections were placed into a 0.1% Triton X solution for 10 min to permeabilize the lipid bilayer, and then treated with a blocking buffer [0.1 M PBS, 5% goat normal serum (GNS), 1% bovine serum albumin (BSA) for 1 h]. Sections were incubated overnight at 4°C in the primary antibody solution containing a 1:750 ratio of 0.1 M PBS to FITC conjugated *Wisteria Floribunda Agglutinin* (WFA, for labeling PNN; Vector Laboratories, Burlingame, CA, USA, FL-135), a 1:10,000 ratio of rabbit anti-PV (Swant, PV25), 1% GNS, 0.5% BSA and 0.1% Tween. After 16–20 h in the primary antibody solution, sections were washed in 0.5% Tween buffer three times for 10 min each. The sections were then incubated in a 1:500 solution of secondary antibody (donkey anti-rabbit, AlexaFluor 647, Life Technologies, Carlsbad, CA, USA, A31573) for 1 h. Sections were washed once with 0.1 M PBS for 5 min and mounted onto glass slides. Vectashield with a DAPI nuclei stain (Vector Labs, Burlingame, CA, USA, F2-135) was used as the mounting medium. Slides were cover-slipped and sealed prior to imaging on a Leica SP5 confocal microscope.

### Image Capture, Analysis and Data Representation

The location of the A1 on coronal sections stained with DAPI was identified using the structure of the hippocampus. This method of identifying mouse A1 has been previously validated with combined electrophysiology and dye placements (Martin del Campo et al., 2012) and cross-referenced with the Paxinos and Franklin, Mouse Brain Atlas (approximately Bregma −2.40 to −3.40) and other studies of the mouse auditory cortex (Cruikshank et al., 2001; Anderson et al., 2009). *Z*-stack images were captured on a Leica SP5 confocal microscope in 2 μM steps. Images were cropped so that the pia and deepest portions of the cortex were visible in a 400 μm wide section, oriented with the pia to the left. Cell counts and intensity measurements were obtained with ImageJ software (NIH). PV+, PNN+ and co-localized (PV/PNN) cells were manually counted in each image. Only PV+ cells containing immunofluorescence throughout the soma, with the exception of the nucleus, met the criteria for being included in the count.

To reduce the potential influence of random and punctate autofluorescence on the PV+ cell counting procedure, images were filtered with the minimum filter function in ImageJ at a setting of two pixels. With this filter, all pixels were replaced with the minimum pixel intensity value in that pixel’s neighborhood (≤2 pixels away). The smallest objects, such as autofluorescent puncta, were erased or nearly erased by the filter, and cell-sized staining areas were slightly reduced in size while maintaining their shape, as only the edges were eroded. This procedure was used for PV stained sections across all age groups.

For PNN analysis, only cells with a clearly formed PNN ring around the soma were included. The PV+ and PNN+ channels were separated for counting in each image. Each cell, either PV+ or PNN+, was marked on their respective image, and the overlap of the two was used to determine the co-localized cell population. Counting bias was avoided by including all cells entirely within the boundaries of the image and those that were in contact with the top border of the image. Those that were in contact with the lower border of the image were excluded from the count. The total area of the cortex was then used to calculate cell densities (cells/mm^2^) of each cell type.

There are layer-specific differences in responses to broadband noise between young and old rat A1 neurons, in a manner suggestive of greater loss of inhibition in superficial than deeper cortical layers (Hughes et al., 2010). Superficial (I–IV) and deep (V–VI) cortical layers are differentially susceptible to loss of PV+ cells in the aging C57 mouse (Martin del Campo et al., 2012). To determine if PNN cell density also declined differentially with depth, the superficial and deep layers were separately analyzed. Layer IV typically terminates at approximately 50% of the depth of the cortex from the pia (Anderson et al., 2009; Martin del Campo et al., 2012), so this value was used to delineate between the two. Any cells contacting the line separating these two cortical sections were included in the layer I–IV cell populations.

In order to determine if the overall cell density changes with age in A1, cell counts were obtained from DAPI stained sections. The cortical column was split into eight sections, each 50 μm wide, and in each image a random number generator was used to determine which of the eight sections to count. This cell density value was multiplied by eight to estimate the total number of cells within the 400 μm wide section of cortex.

Deterioration of PNN may manifest as changes in intensity without a loss of PNN+ cell density (Enwright et al., 2016). To determine PNN intensity throughout A1 and at the single cell level, images were converted into 8-bit format (where 0 is total absence of PNN). The average intensity of the cortex was determined with the “Plot Profile” function in ImageJ. To accomplish this, a rectangle (width of 400 μm and depth extending from pia to end of layer VI) was drawn on the cortical section. Running the plot profile function on this rectangle yields a plot of depth through the cortex vs. average gray scale intensity at that depth. These intensity values were averaged to obtain an average PNN intensity for an individual image. To determine if intensity was different between superficial and deep layers, all values from the first half of this data set were averaged for superficial and the second half was averaged for deep layers.

We also developed a method to analyze PNN intensity around individual cells. PNN intensity at the cellular level was determined by cropping a 40 μm^2^ square region centered on randomly selected PNN+ cells. Each cell was assigned a number and a random number generator was used to determine which of the cells would be analyzed. Fifteen cells in each image were randomly chosen for intensity analysis. The intensity was plotted as a function of the distance along the midline of the 40 μm^2^ region using the Plot Profile function (ImageJ). This resulted in a bimodal peaked scatter plot with the two peaks corresponding to the locations where the line intersected the most intense part of the PNN ring structure on both sides of the cell (e.g., Figure 7A). In order to account for possible differences in cell size, a custom Matlab script was used to find the pixel at which the intensity drops to 90% of the peak values on either side of the cell. The intensity values within these two cutoff points were averaged for each cell and every cell average within an image was averaged to obtain a single value of PNN+ cell intensity per image. These data were used in the statistical tests. In order to account for differences in background intensity, a 40 μm^2^ square was placed in layer I of A1 which is devoid of PNN. The average value of this region was then subtracted from all PNN intensity measurements before making final comparisons.

### Data Analysis

For the PV, PNN and PV/PNN cell density and across-A1 PNN intensity analyses, each image contributed a single value for each measure. The means of these values were compared across age using One Way ANOVA with *post hoc* pairwise comparisons. We consider sections drawn from an individual mouse to be independent samples because the sections are a minimum of 160 μm from each other and therefore likely cover parts of A1 with different response selectivity. For the cell-specific PNN intensity analysis, each image provided an average intensity value from up to 15 randomly selected cells (see above). The means of these values were compared across age using One Way ANOVA and *post hoc* pairwise comparisons.

Table 1 provides the details on each mouse’s age (column 2), strain (column 3) and the number of sections on which IHC was performed (column 4). An outlier analysis was done on the available images based on PV and PNN cell densities. Outliers were identified as images in which the values for cell density was above the value given from the equation, Q3 + IQR * 1.5 or below the value given from equation. Q1 − IQR * 1.5, where Q1 is the cutoff of the first quartile of the data set range, Q3 is the cutoff of the third quartile of the dataset, and IQR (Interquartile Range) = Q3 − Q1 (Weber et al., 2001; Leys et al., 2013). Based on this analysis, the following PNN IHC images were excluded: three images (out of 34) from the old C57 dataset, one image (out of 25) from the young C57 group, three images (out of 14) from the middle aged CBA group and two images (out of 23) from the O CBA group. The right-most column of Table 1 indicates the number of images that were used for the PNN statistical analyses. None of the PV images were excluded.

**Table 1:** **Details on age, strain and number of sections stained**.

Mouse	Age (months)	Strain	All sections	PNN sections
PM007	2	C57	4	4
PM008	2	C57	4	4
PM011	2	C57	4	4
PM034	3	C57	5	5
PM035	3	C57	4	3
PM036	3	C57	4	4

PM006	7	C57	4	4
PM025	8	C57	2	2
PM037	6	C57	5	5
PM038	6	C57	5	5
PM039	6	C57	4	4

PM001	13	C57	3	3
PM002	13	C57	3	3
PM003	13	C57	5	5
PM026	24	C57	4	4
PM027	24	C57	4	4
PM028	24	C57	4	3
PM046	14	C57	4	2
PM048	14	C57	7	7

PM052	1	CBA	5	5
PM053	1	CBA	4	4
PM054	2	CBA	4	4
PM055	2	CBA	3	3
PM056	2	CBA	2	2
PM057	2	CBA	3	3
PM058	2	CBA	3	3

PM029	6	CBA	2	2
PM030	6	CBA	2	2
PM031	6	CBA	5	3
PM032	6	CBA	3	3
PM033	6	CBA	2	1

PM040	15	CBA	3	3
PM041	15	CBA	4	2
PM042	15	CBA	4	4
PM043	15	CBA	4	4
PM044	15	CBA	4	4

*From left to right, the columns indicate the mouse identification, age in months, strain, the number of sections stained and the number of sections used for PNN data analysis following outlier analysis*.

It can also be noted from Table 1 that in terms of age groups, the Y and the M were relatively well matched between the two strains. However, in the O group, three out of eight C57 mice were 24 month old. All other O group mice were in the 13–15 month range. Due to the possibility that the C57 data may be inflated by the 24 month old mice, a separate analysis was done in which the 24 month old C57 mice were excluded. The major conclusions presented below in the “Results” Section regarding age-related changes in the C57 mice were unchanged with the removal of the 24 month old mice. Therefore, the “Results” Section and figures to follow first present data from all the mice shown in Table 1. The last paragraph of the “Results” Section presents the analyses with the 24 month old C57 mice excluded.

### Statistical Analyses

All data sets were compared across age (three levels, Y, M, and O) and within strain (either C57 or CBA). All statistical tests performed were One Way ANOVAs, unless stated otherwise. Typical One Way ANOVA was used when data were distributed normally and the Kruskal-Wallis One Way ANOVA on Ranks was used if data were not normally distributed, according to the Kolmogorov-Smirnov test. Statistical tests are reported with the F (ANOVA) or H (Kruskal-Wallis) for main effect of age. All pairwise multiple comparison procedures (Tukey test for the One Way ANOVA and Dunn’s method for the Kruskal-Wallis test) were run subsequent to finding a main effect (*p* < 0.05) in a data set. Multiple comparisons procedures statistics are reported as *p*-values for a specific comparison of age groups (e.g., Y-O: *p* < 0.05).

## Results

Reduced inhibition is a consistent feature observed in sensory cortices of aging animals. To test the hypothesis that the cellular mechanisms of age-related reduction in inhibition includes the PV/PNN system, the main goal of this study was to examine PV+, PNN+ and PV/PNN co-localized cell density in A1 of two strains of mice (C57 and CBA) across three different age groups.

### PNN Cell Density Declines With Age in A1 of Both C57 and CBA Mice

**Figure 1:**
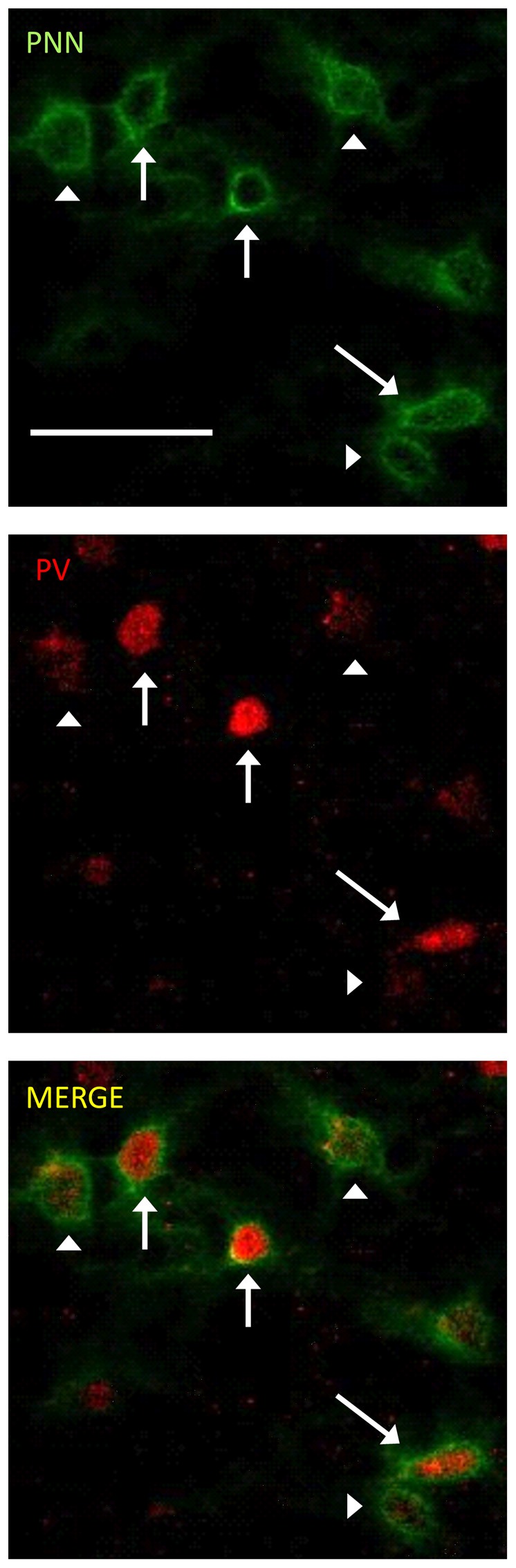
**Representative examples of perineuronal net (PNN+) and parvalbumin (PV)/PNN co-localized cells are shown.** PNN+ cells (green channel) were identified by their complete or near complete ring structure, with relatively less intense staining in the area corresponding to the soma. PV+ cells (red channel) were identified by a staining pattern throughout most of the soma. PNN+ cells without PV are marked with arrowheads. PV+/PNN+ co-localized cells are indicated with a solid arrow. These labels are preserved when the images are separated out into the two channels and when the images are merged. The scale bar is 50 μm and applies to all images.

**Figure 2:**
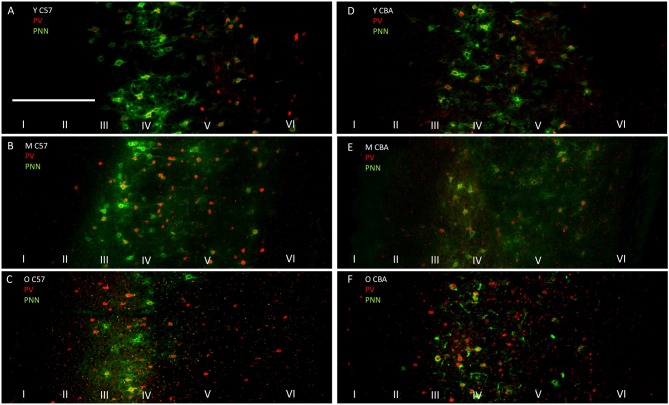
**Representative photomicrographs through primary auditory cortex (A1) from Young (A), Middle (B) and Old C57 mice (C) and Young (D), Middle (E) and Old CBA (F) mice.** The scale bar in **(A)** is 250 μm and applies to all images.

The photomicrographs in Figure 1 illustrate the cells that were counted as PNN+ or PV/PNN co-localized cells. Each solid arrow or arrowhead points to a PNN+ with a complete or near complete ring structure. Arrowheads are PNN+ cells without PV+ immunofluorescence. The arrows in the merged image point to PV/PNN co-localized cells. The arrowheads in the merged image point to cells that were counted as PNN+. Qualitative observations of staining patterns show that PNN+ cells are found throughout A1, with a banding pattern of PNN+ cells and diffuse neuropil PNN staining in layer IV (Figure 2). Generally, more PNN+ cells were observed in layers IV-VI compared to layers I-III. PNNs frequently appeared around PV+ cells, especially within the layer IV band. However, a number of PNN+ cells were devoid of PV staining and *vice versa*. These observations were consistent across the mouse strains and age (see Figure 2) and with previous studies of PNN expression in young mouse A1 (Happel et al., 2014; Fader et al., 2016).

**Figure 3:**
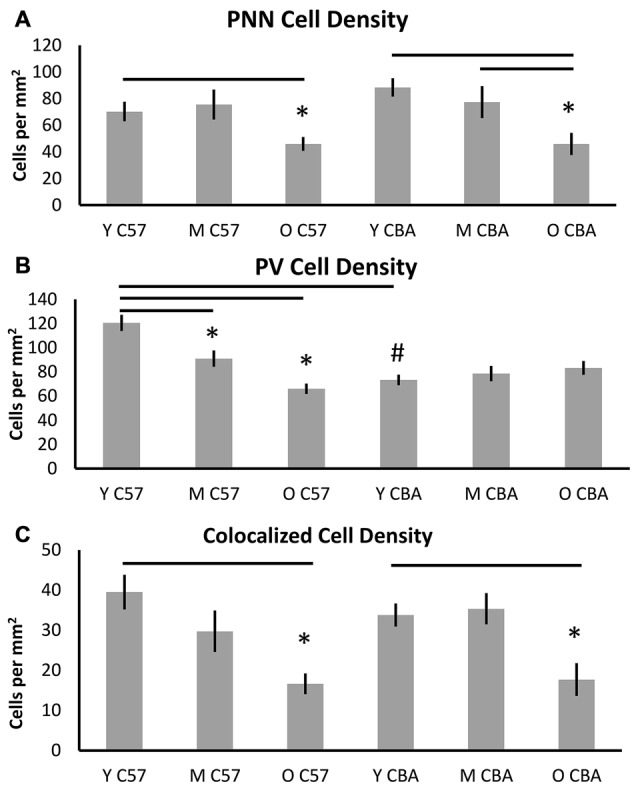
**Density (cells per mm^2^) of (A) PNN+, (B) PV+, and (C) PV/PNN co-localized cells in A1 (all six layers combined) are shown.** Asterisks indicate a significant difference from the Y group within the same strain. The “#” indicates a significant difference between age-matched groups across strains (*p* < 0.05).

Figure 3 shows PNN+, PV+ and PV/PNN co-localized cell density in A1 when all six layers of the cortex were considered. The PNN+ cell density decreased significantly with age in A1 of both the C57 mouse strain (Figure 3A, *H*_(2,76)_ = 9.026, *p* = 0.011) and CBA strain (Figure 3A, *F*_(2,58)_ = 18.286, *p* < 0.001). In the C57 group, the difference was only significant between Y and O mice (Tukey: Y-O *p* < 0.05). In the CBA mouse, PNN+ cell populations significantly decreased between Y vs. O and M vs. O (Dunn: Y-O, M-O *p* < 0.05), but not between Y vs. M (Dunn: Y-O *p* = 0.183).

In contrast to the decline of PNN+ cell density in both strains, PV+ cell density declined with age only in the C57 mouse (Figure 3B). The PV+ cell density declined with age in C57 mice (*F*_(2,76)_ = 24.547, *p* < 0.001) and the decrease was significant between each age group (Tukey: Y-M: *p* = 0.004, Y-O: *p* < 0.001, M-O: *p* = 0.010), but not in the CBA mice (*F*_(2,58)_ = 0.993, *p* = 0.377). There was a significant age-related decline in PV/PNN co-localized cell density in both strains (Figure 3C; C57: *H*_(2,76)_ = 18.558, *p* < 0.001; CBA: *H*_(2,58)_ = 16.018, *p* < 0.001). This difference was observed only in the Y vs. O comparison (C57: Dunn: Y-O: *p* < 0.05; CBA: Dunn: Y-O: *p* < 0.05). We further compared the PV+ cell density between the young mice of each strain. Interestingly, there were more PV+ cells in the Y C57 than Y CBA mice (Mann Whitney Rank Sum *t*-Test, *U*_(47)_ = 524, *p* < 0.001). This indicates strain-specific differences in PV expression in young mouse A1.

There were no age-related differences in the total number of cells based on DAPI staining for either mouse strain (C57: *F*_(2,76)_ = 2.381, *p* = 0.106; CBA: *H*_(2,58)_ = 3.606, *p* = 0.165). This indicates that the observed decline in PV and PNN is not due to non-specific changes in cell density with age. Taken together, these data indicate strain and age specific differences in PV and PNN expression in A1. Consistent across both strains was the observed age-related decline in PNN cell density and PV/PNN co-localized cell density in A1.

### Deep and Superficial Layers were Similarly Susceptible to Age-Related PNN Deterioration

**Figure 4:**
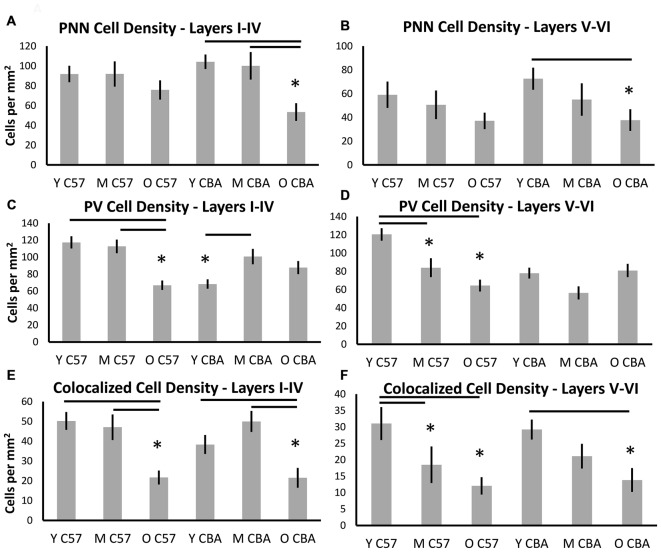
**Density (cells per mm^2^) of (A,B) PNN+, (C,D) PV+, and (E,F) PNN+/PV+ cell types in superficial layers (layers I–IV) and deep layers (layers V–VI) of the auditory cortex.** Asterisks indicate a significant difference from the Y group within the same strain (*p* < 0.05).

Although some exceptions were seen, the general observation was that both superficial and deep layers were similarly susceptible (Figure 4). In the C57 mice, the significance in PNN density changes was lost when the data were sub-divided into the layers. However, the trend towards reduction is preserved (Figures 4A,B, Layers I–IV: *H*_(2,76)_ = 4.1, *p* = 0.12. Layers V–VI: *H*_(2,76)_ = 4.629, *p* = 0.09). In the CBA mice, data from all cortical layers showed a reduction in PNN cell density with age (Figure 3). This difference was also seen in the layer specific analysis of A1 (Figures 4A,B, Layers I–IV: *F*_(2,58)_ = 9.453, *p* < 0.001; Layers V–VI: *F*_(58,2)_ = 9.96, *p* = 0.007). The decrease in both layer sub-divisions was apparent in the old age group (Layers I–IV: Tukey: Y-O, M-O: *p* < 0.05; Layers V–VI: Tukey: Y-O: *p* < 0.05).

Layer specific analysis of PV+ cell density in C57 mice showed an age-related decline in both superficial (Layers I–IV: *F*_(2,76)_ = 16.917, *p* < 0.001), and deep layers (Layers V–VI: *F*_(2,76)_ = 16.525, *p* < 0.001; Figures 4C,D). In the superficial layers, this reduction in PV+ density was seen in the O mouse group (Tukey: Y-O, M-O: *p* < 0.05; Figure 4C), whereas the reduction in the deep layers occurred earlier, appearing in the M group (Tukey: Y-O, Y-M: *p* < 0.05; Figure 4D). There was no age-related change in PV+ cell density in the CBA mice in either the superficial or deep layers. Counter-intuitively however, there was a significant increase from young to middle age in PV+ cell density in the superficial layers (Figures 4C,D, Layers I–IV: *H*_(2,58)_ = 7.801, *p* = 0.020; Dunn: M-Y: *p* < 0.05; Layers V–VI: *H*_(2,58)_ = 5.613, *p* = 0.06).

Finally, layer-specific PV/PNN co-localization was characterized in both mouse strains. The decline in PV/PNN co-localized cell density that appears across cortical layers in O C57 and O CBA mice is present in both the superficial and deep cortical layers (Figures 4E,F, C57: Layers I–IV: *F*_(2,76)_ = 11.502, *p* < 0.001; Tukey: Y-O, M-O: *p* < 0.05; Layers V–VI: *H*_(2,76)_ = 7.519, *p* = 0.023; Dunn: Y-O, Y-M: *p* < 0.05. CBA: Layers I–IV: *F*_(2,58)_ = 7.216, *p* = 0.002; Tukey: Y-O, M-O: *p* < 0.05; Layers V–VI: *F*_(2,58)_ = 5.607, *p* = 0.006; Tukey: Y-O: *p* < 0.05). Taken together, these data reveal an overall similarity between layers I–IV and layers V–VI in their susceptibility to age-related changes in PV and PNN cell densities. Some exceptions to this rule include the earlier susceptibility of C57 deep layers to PV+ cell decline and the increase in PV+ cell density in superficial layers in CBA mice.

### PNN Intensity Analysis

**Figure 5:**
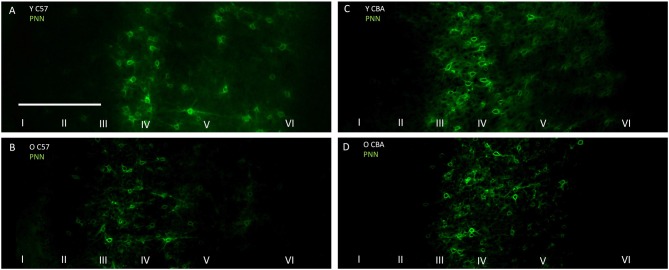
**Representative examples of PNN images used for analyzing labeling intensity at the whole A1 level. (A,B)** Show young and old C57 mouse PNN examples, respectively. **(C,D)** Show young and old CBA PNN staining examples, respectively. The scale bar in **(A)** is 250 μm and applies to all images.

**Figure 6:**
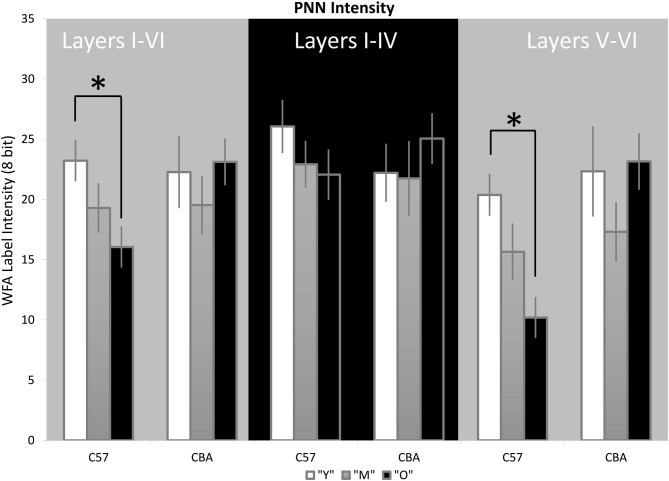
**PNN staining intensity declines in A1 in C57, but not CBA, mice.** The average Wisteria Floribunda Agglutinin (WFA) intensity is shown for the entire depth of A1 (left set of bars), superficial layers (middle) and deep layers (right). Asterisks indicate significant difference (*p* < 0.05).

Previous studies suggest that a decline in PNN intensity may reflect changes in PNN organization, and due to their role in buffering ions and stabilizing synaptic contacts, may lead to changes in excitation/inhibition balance. This change in PNN intensity may occur independent of changes to overall PNN+ cell density (Carulli et al., 2010). Therefore, we analyzed PNN intensity in A1. Representative images of PNN staining are shown in Figure 5 to illustrate the age-related decline in PNN staining. In the C57 mice, the average PNN intensity is reduced for the Y-O comparison (Figure 6, *H*_(2,76)_ = 9.619, *p* = 0.008; Y-O: *p* < 0.05). Layer specific analysis showed that this age-related reduction in PNN intensity is only observed in the deep cortical layers of O C57 A1 (Layers I–IV: *F*_(2,76)_ = 1.040, *p* = 0.346; Layers V–VI: *H*_(2,76)_ = 18.360, *p* < 0.001, Dunn: Y-O: *p* < 0.05). Conversely, in the CBA mouse A1, there was no significant difference in PNN intensity with age (Figure 6; *H*_(2,58)_ = 2.060, *p* = 0.357). Comparison of PNN cell density vs. PNN intensity (Figures 4, 6) indicate that in C57 mouse A1, there is a decline in PNN+ cell density and the remaining PNN+ cells show reduced intensity. On the other hand, in the CBA mouse A1, there is a decline in PNN+ cell density, but the remaining PNN+ cells do not show a significant change in PNN staining intensity.

**Figure 7:**
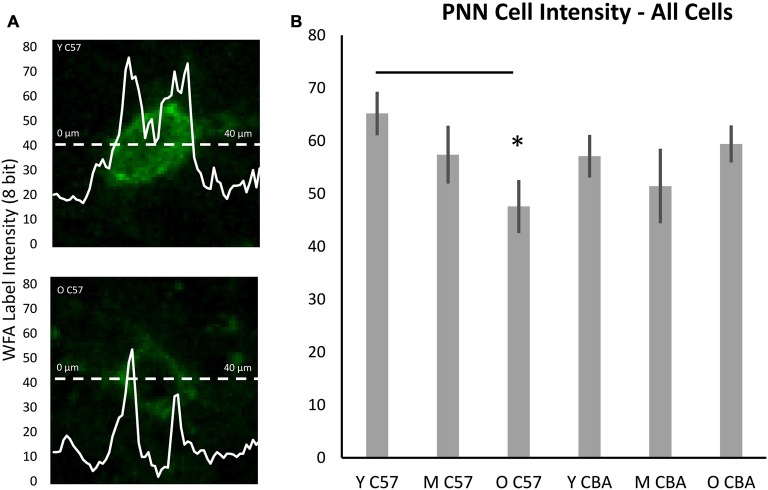
**Cellular PNN intensity analysis. (A)** Example images for analysis of PNN intensity around a cell. The dashed line represents the points at which the PNN gray scale intensity was measured. The double peaked curve shows the intensity at each point on the dashed line. In **(A)**, the upper image is from a young C57 mouse and the bottom image is from an old C57 mouse. These examples illustrate the decline in cellular PNN intensity shown in **(B)**. **(B)** Average of all cellular PNN intensity plots for each age group and strain. Asterisks indicate a significant difference from the Y group (*p* < 0.05).

While the above analysis provides information about PNNs across the entire depth of A1, studies of epileptogenesis and songbird brain development (Dityatev et al., 2007; Balmer et al., 2009) have suggested the integrity of PNN around the cell may provide more subtle markers of changes to PNN. Therefore, we developed a method to analyze PNN intensity in individual cells and compared A1 neurons across the three age groups. Analysis of PNN intensity at the individual cell level showed data that were consistent with the age-related changes in the average PNN intensity across all cortical layers. That is, there was a decrease in average single cell intensities in the C57 A1 (Figure 7B; *F*_(2,76)_ = 3.559, *p* = 0.034) and the difference is significant between Y and O C57 mice (Tukey: Y-O: *p* < 0.05). No age-dependent change is observed in CBA A1 average cellular PNN intensity (Figure 7B; *H*_(70,2)_ = 4.937, *p* = 0.085).

**Figure 8:**
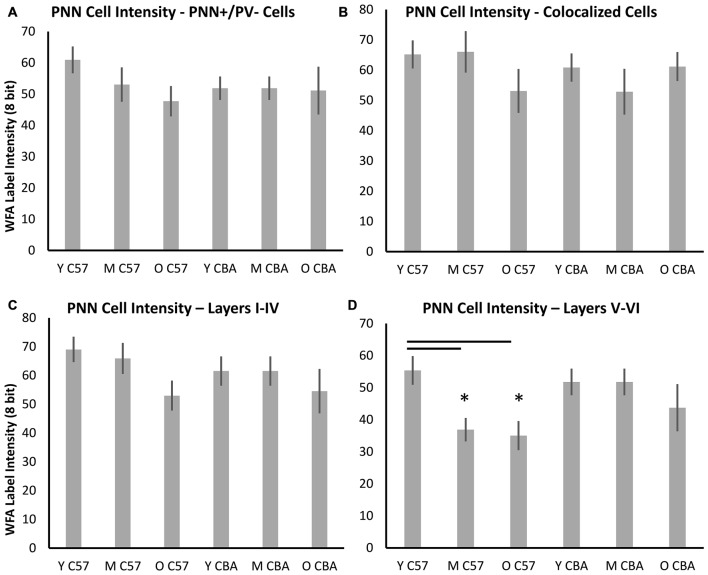
**Changes in cellular PNN intensity in specific types of PNN cells. (A)** Cellular PNN intensity in PNN+ cells that do not co-localize with PV. **(B)** Cellular PNN intensity in PNN+ cells that co-localize with PV. **(C)** Cellular PNN intensity in PNN+ cells in layers I–IV of A1. **(D)** Cellular PNN intensity in PNN+ cells in layers V–VI of A1. Asterisks indicate a significant difference (*p* < 0.05) from the Y group.

PNNs form most prominently around PV+ cells in A1, but they also form around other cells, which do not express PV. Previous studies have shown that PV cells with enwrapped PNNs are less susceptible to oxidative stress (Cabungcal et al., 2013). Age-related changes in PV and PNN expression may occur in specific subsets of cells that are identifiable with PV and/or PNN. Therefore, we quantified PNN intensity across the following cell types (Figure 8): (A) PNN+ cells with no PV, (B) PNN cells with PV, (C) PNN+ cells in layers I–IV of A1, and (D) PNN+ cells in layers V–VI of A1. The decline in average PNN+ cell intensity with age in the C57 strain is lost when subdividing PNN+ cells into PV+ or PV− subpopulations (Figures 8A,B, PV+ PNN: *F*_(2,76)_ = 1.34, *p* = 0.269; PV− PNN: *F*_(2,76)_ = 1.902, *p* = 0.157). When subdividing PNN+ cells into superficial and deep layer cells, there was an age-related decline in the average cellular PNN intensities of cells in both depths (Figures 8C,D, Layers I–IV: *F*_(2,76)_ = 3.096, *p* = 0.051; Layers V–VI: *F*_(2,76)_ = 5.355, *p* = 0.007; Tukey: Y-M, Y-O: *p* < 0.05). No significant differences in PV+/PNN (*F*_(70,2)_ = 0.61, *p* = 0.547) or PV−/PNN (*H*_(70,2)_ = 4.152, *p* = 0.125) cell intensities were observed between age groups in CBA mice. Likewise, no significant differences in PNN+ cell intensity were observed for cells residing in Layers I–IV (*F*_(70,2)_ = 0.531, *p* = 0.591) or in Layers V–VI (*H*_(70,2)_ = 5.079, *p* = 0.079) between age groups in the CBA mouse strain. This was to be expected given that there was no difference in average PNN cell intensities with age in the CBA mice (Figures 6, 7).

Given that there were three 24 month old C57 mice, but no CBA mice in that age range, there was a possibility our findings were influenced by the relative difference in age ranges between the two “O” groups (Table 1). To account for this, the analyses were performed with the 24 month old animals excluded from the O C57 group. All main effects of age in the C57 strain remained. Specifically, no changes were observed in the decline of PNN+, PV+, or PV/PNN co-localized cell densities with age in C57 (PNN+: *H*_(2,64)_ = 8.077, *p* = 0.018; PV+: *F*_(2,64)_ = 23.479, *p* < 0.001; Co-localized cells: *H*_(2,64)_ = 16.829, *p* < 0.001). The effect of age on PNN intensity at the level of the entire A1 column and at the individual cell level also remained significant (Layers I–VI: *H*_(2,64)_ = 16.120, *p* < 0.001; PNN cell intensity: *F*_(2,64)_ = 9.578, *p* < 0.001). The only change observed by removing the 24 month old C57 mice was in the statistical difference observed in the PNN cell intensities of M and O C57 mice that was not seen when the oldest C57 mice were included (Tukey: *p* < 0.05 for Y-O C57 and for M-O C57 comparisons).

## Discussion

The main findings of this study in relation to aging are summarized in Table 2. We show that PNN+ and PV/PNN co-localized cell densities decline with age in A1 of both C57 and CBA mice. PNN staining intensity was also reduced in the C57 mouse A1. The decline in PV+ cell density in aging C57 mice was consistent with a previous study (Martin del Campo et al., 2012) but there was no change in PV+ cells density in CBA mice.

**Table 2:** **Summary of the main changes in expression of PV and PNN in A1 of C57 and CBA mice**.

Analysis type	C57 Young → Old	CBA Young → Old
PNN+ cell density	Decrease	Decrease
PV+ cell density	Decrease	No change
PV/PNN co-localized cell density	Decrease	Decrease
Average PNN intensity across layers	Decrease	No change
Average PNN cellular intensity	Decrease	No change

*Comparisons are only shown for all layers of A1 and only between young and old mice. Layer-specific changes and middle-aged data are omitted for clarity and can be found in the “Results” Section*.

### PNN Expression in Young Mouse A1

Although the expression of PNN and its association with specific cell types have been well characterized in rodent visual and somatosensory cortex (Pizzorusso et al., 2002; McRae et al., 2007; Liu et al., 2013; Takesian and Hensch, 2013) and subcortical auditory areas (Beebe et al., 2016), the expression patterns in A1 are only beginning to be described (reviewed in Sonntag et al., 2015). The relationship between PV and PNN expression in A1 has not been previously characterized. Consistent with previous articles on mouse A1, PNN expression was seen in layers II–VI, with a particularly high concentration in layer IV (Happel et al., 2014; Fader et al., 2016). The PNN+ cell density was similar in C57 and CBA mice. A strong association between PV+ and PNN+ cells in A1 was seen as reported in a number of other brain regions (Kosaka and Heizmann, 1989; Celio, 1993; Pantazopoulos et al., 2006; Liu et al., 2013; Yamada et al., 2015) is also present in A1. Approximately 35% of the PV+ cells in the C57 mouse A1 and 50% of PV+ cells in the CBA mouse A1 were also PNN+ (ratio of data in Figures 2B,C). Future studies are needed to classify the various types of neurons that express PNN in A1.

### Age-Related Changes in PNN and PV Expression

The main result of the current study is that PNN deteriorates in A1 with age. As far as we are aware, this is the first study to examine age-related changes in PNN expression in sensory cortex. PNN deterioration with age manifested as both a reduction in PNN+ cell density in C57 and CBA mice, and decreased PNN intensity across A1 and around cell bodies in C57 mice. Layer-specific comparisons do not provide support for deep vs. superficial layers being more or less susceptible. In both strains, the PV/PNN co-localized cell density also declines significantly with age. The percentage of PV+ cells that also express PNN drops to ~25% in old mice of both strains. Age-related decline of PV+ cells was observed only in the C57 mice. The PV+ changes are analogous to those observed in aging rats (Ouda et al., 2008). The Long-Evans rat (normally aging) did not show a loss in PV+ cell density in A1 with age while the Fisher F344 rat (presbycusis) showed a significant loss. Together, the PV/PNN system appears to be more susceptible in the C57 mice A1 compared to the CBA mice. The major age-related trends in the PV/PNN data were not affected regardless of whether the three oldest (24 months) C57 mice were included or not. Although the sample size is small, this suggests that much of the PV/PNN change in C57 mice occurs before 14 months of age. Henry and Chloe (1980) showed that most of the threshold shifts and hair cell loss in C57 mice occur by 15 months of age. Further loss in 25 month old C57 mice was relatively small. Willott (1986) showed a similar trend for inferior colliculus tuning curve thresholds with most of the change seen by 14 months. On the other hand, CBA mice younger than 15 months were similar to young mice in thresholds and hair cell counts. With the caveat that these may simply be strain-specific differences, these data suggest that hearing loss that occurs between 2–14 months in the C57 mice exacerbates age-related changes in inhibitory systems in A1. Future studies will investigate this suggestion more directly by controlled induction of hearing loss during aging in the CBA mouse.

PNNs are highly structured extracellular matrix components around cell bodies and proximal dendrites. Components of PNN include CSPGs, hyaluronan, tenascin-R, link proteins, Reelin and semaphorin 3A. The main CSPGs in PNN are aggrecan, neurocan, brevican, versican and phosphacan. This study used the lectin WFA to stain PNN because it is the most widely used method to study PNN expression with an extensive literature that supports the specificity of WFA for CSPGs. WFA binds specifically to N-acetyl-D-galactosamine. However, the specific CSPGs detected by WFA are unclear. Therefore, the interpretation that PNN deteriorates with age must be considered with the caveat that the results may be due to a change in the composition of PNN detected by WFA. Likewise, the reduction in PNN intensity seen in C57 A1 may reflect changes in CSPG protein levels and composition and/or hyaluronan synthase levels.

### Mechanisms and Implications for Auditory Function with Presbycusis

PV and PNN deterioration with age may result in abnormal synaptic regulation and firing properties of PNN-ensheathed PV+ neurons (Lucas et al., 2010; Berretta et al., 2015). PNNs surround mostly GABAergic neurons in sensory cortex with preference for PV+ neurons. PNN deterioration may leave PV+ cells vulnerable to damage oxidative stress damage (Cabungcal et al., 2013). PV+ cells are susceptible to oxidative stress (Powell et al., 2012). This suggests that the deterioration of PNN with age may impact auditory function by contributing to decreasing cortical inhibition mediated by PV+ cells. This notion is supported by findings of Shah and Lodge (2013) who showed that degradation of PNN enhances hippocampus activity. The PV+ cells may shape inhibitory components of receptive fields and responses to rapid spectrotemporal cues (Atencio and Schreiner, 2008; Wu et al., 2008). Reduced inhibition may underlie spectrotemporal processing and speech recognition deficits with age (Walton et al., 1998; Mendelson and Ricketts, 2001; Razak and Fuzessery, 2009; Parthasarathy and Bartlett, 2011; Suta et al., 2011; Trujillo and Razak, 2013).

Strong evidence also suggests that PNNs provide stability to the excitation-inhibition balance during development and that adult plasticity can be promoted by breaking down PNNs (Takesian and Hensch, 2013). The deterioration of PNN with age may open up the cortical circuit to altered excitation-inhibition balance. The age-related decline in PNN density (C57 and CBA), intensity (C57) and PV cell density (C57) seen in the present study may result from decreasing afferent excitation with age and hearing loss and underlie a compensatory decrease in inhibition. Such reduced inhibition with age may be one of the steps in causing an increase in cortical gain and potentially, pathological activity (e.g., tinnitus). Evidence for such pathological activity correlated with changes in PNN comes from studies of epileptogenesis and schizophrenia (Dityatev et al., 2007; McRae et al., 2012).

While the co-occurrence, and age-related loss, of PV and PNN have been discussed above primarily from the view of reduced inhibition, alternate explanations are possible. One is that the loss of PNN actually increases excitability of PV+ inhibitory neurons (Dityatev et al., 2007). From this perspective, age-related decline of PV in GABAergic neurons in auditory cortex occurs independent of PNN changes. The loss of PNN may then be a homeostatic mechanism that compensates for altered PV neuron function. At present, it is still unclear how PNN contributes to PV/GABA neuron function. The alternate hypotheses proposed above could be addressed by future electrophysiology studies that compare activity of PV neurons with and without PNN and compare activity of PV/PNN neurons before and after enzymatic degradation of PNN.

The events leading up to PNN deterioration with age may include changes to matrix metalloproteases (MMP) and cartilage link proteins (e.g., Ctrl1). MMP-9 is an endopeptidase that cleaves extracellular matrix including PNN (Ethell and Ethell, 2007; Reinhard et al., 2015). MMP-9 levels are regulated by activity and high MMP-9 levels lead up to increased breakdown of PNN. This suggests the hypothesis that MMP-9 levels increase with age. Carulli et al. (2010) showed that mice lacking Ctrl1, a PNN component, show attenuated PNNs including reduced intensity. The attenuated PNN promoted cortical plasticity in adults. Thus, future studies of aging A1 will analyze expression levels of MMP-9 and Ctrl1 to identify the various players in age-related decline in auditory function and identify potential therapeutic avenues to delay or prevent the decline.

## Conclusion

These data show for the first time that PNN declines in aging sensory cortex. These changes likely contribute to altered inhibitory neurotransmission, which can then lead to impaired spectrotemporal processing. In humans, this leads to impaired speech recognition abilities. An age-related decline in PNN cell density is present in both the C57 and CBA A1, but a decrease in PNN intensity is seen only in the C57 mice suggesting a possible exacerbation with hearing loss in addition to aging. Future studies will examine the specific proteoglycans and other components of PNN that are susceptible to aging and whether an up-regulation of MMP-9 precedes the changes in PNN. Future studies should investigate if age-related decline in PNN density and intensity is present in other sensory cortical regions.

## Author Contributions

DHB and KAR designed the experiments and wrote the article. DHB and JK performed the experiments. DHB, JK, OJ, ERP and KAR analyzed the data.

## Conflict of Interest Statement

The authors declare that the research was conducted in the absence of any commercial or financial relationships that could be construed as a potential conflict of interest.
